# Comparing the efficacy of low-load resistance exercise combined with blood flow restriction versus conventional-load resistance exercise in Chinese community-dwelling older people with sarcopenic obesity: a study protocol for a randomised controlled trial

**DOI:** 10.1186/s12877-023-04592-9

**Published:** 2023-12-19

**Authors:** Min Zhuang, Jinli Shi, Jian Liu, Xiangfeng He, Nan Chen

**Affiliations:** 1 Department of Rehabilitation, Chongming Hospital Affiliated to Shanghai University of Medicine and Health Sciences, Shanghai, China; 2 School of Exercise and Health, Shanghai University of Sport, Shanghai, China; 3 Community Health Service Center of Chengqiao Town, Chongming District, Shanghai, China; 4 Community Health Service Center of Gangxi Town, Chongming District, Shanghai, China; 5 Department of Rehabilitation, Xinhua Hospital Affiliated to Shanghai Jiaotong University School of Medicine, Shanghai, China

**Keywords:** Sarcopenic obesity, Resistance exercise, Blood flow restriction

## Abstract

**Introduction:**

Sarcopenic obesity (SO) is characterised by decreased muscle mass, diminished muscle strength and/or reduced physical performance and a high percentage of body fat (PBF). Conventional-load resistance exercise (CRE) may be difficult for older people with SO owing to their declining physical functions. Low-load resistance exercise (LRE) combined with blood flow restriction (BFR; LRE-BFR) is a viable alternative to CRE for improving muscle mass and strength and potential exercise mode for managing SO. This study has two objectives: (1) to comprehensively evaluate the efficacy of CRE and LRE-BFR in improving body composition, muscle strength, physical performance, haematological parameters, cardiovascular disease (CVD) risk factors and quality of life and (2) to compare the efficacy of CRE and LRE-BFR and explore their potential mechanisms.

**Methods and analysis:**

This work is a 12-week assessor-blinded randomised clinical trial that will be conducted thrice a week. Sarcopenia will be defined using the Asian Working Group for Sarcopenia 2019, and obesity will be determined using the criteria developed by the World Health Organization. Community-dwelling older people aged ≥ 65 years will be screened as the participants using inclusion and exclusion criteria. A total of 33 participants will be randomised into a CRE group (*n* = 11), an LRE-BFR group (*n* = 11) and a control group that will be given only health education (*n* = 11). The primary outcomes will be knee extensor strength and PBF, and the secondary outcomes will be body composition, anthropometric measurements, muscle strength of upper limbs, physical performance, haematological parameters, CVD risk factors and quality of life. The outcomes will be measured at the baseline (week 0), end of the intervention (week 12) and follow up (week 24). All the collected data will be analysed following the intention-to-treat principle.

**Ethics and dissemination:**

The Ethics Research Committee has approved this study (approval No. CMEC-2022-KT-51). Changes or developments in this study will be reported at www.chictr.org.cn.

**Trial registration:**

ChiCTR2300067296 (3 January 2023).

**Supplementary Information:**

The online version contains supplementary material available at 10.1186/s12877-023-04592-9.

## Introduction

The accompanying features of sarcopenia and obesity include sarcopenic obesity (SO), which is characterised by decreased skeletal muscle mass, diminished muscle strength and/or reduced physical performance and a high percentage of body fat (PBF) [1]. A meta-analysis of 50 studies showed that the global prevalence of SO was 11% in December 2020, meaning that more than 1 in 10 older people demonstrated adverse health outcomes globally [2]. Several studies reported that SO may result in a two- to threefold higher risk of functional disability [3] as well as hypertension [4], hyperglycaemia [4], dyslipidaemia [5], type 2 diabetes [6] and insulin resistance (IR) [5] compared with sarcopenia alone or obesity alone. The rise in cardiovascular disease (CVD) risk factors has been identified as one of the most important public health problems in the world [7], and CVD is the leading cause of death worldwide [8]. Stephen et al. [9] indicated that older people with SO have an increased risk of CVD by 23% compared with those without SO. The negative health outcomes of older people with SO indicate that early identification and effective intervention are necessary to improve their quality of life.

The pathogenesis of SO is multifactorial. The common pathophysiological basis of sarcopenia and obesity may play a synergistic role in the progression of SO, including increased inflammatory factors (e.g. interleukin 6 [IL-6], C-reactive protein [CRP] and tumour necrosis factor-α [TNF-α]), IR (e.g. serum insulin), endocrine dysfunction (e.g. insulin-like growth factor 1 [IGF-1], leptin [LEP] and adiponectin [ADP]), changes in myostatin (MSTN) and decreased physical activity (PA) [10–12]. In the absence of specific pharmacological treatment, PA is considered to be an effective strategy for managing SO [13, 14].

Specific PA guidelines for older people with SO are lacking. Conventional-load resistance exercise (CRE) is essential for increasing muscle mass and strength and improving physical performance and plays a positive role in reducing PBF and thus may be a promising strategy for managing SO [15–17]. Unfortunately, studies on the efficacy of CRE in managing SO are limited. To increase muscle mass and muscle strength, the American College of Sports Medicine (ACSM) recommends an exercise load of 60%–80% of one-repetition maximum (1RM) for older people [18]. However, this load may induce excessive stress on the joints and connective tissues of older people, which may increase their risk of injury [19]. CRE depends generally on gym machines, which are expensive and not portable, thereby making them unsuitable for most community settings [20]. Therefore, finding an alternative to CRE that is cheap, portable and has a low risk of injury is essential to manage SO.

Blood flow restriction (BFR) is a technique that involves the use of a bandage to apply pressure to a proximal limb to block venous blood return and partially block arterial blood flow, thereby increasing the local metabolic pressure of the body [21, 22]. Lixandrão et al. [23] validated that the combination of low-load resistance exercise (LRE) with BFR (LRE-BFR) at 20%–30% of 1RM is a viable alternative to CRE for improving muscle mass and strength. Similarly, a recent systematic review and meta-analysis explored the effects of BFR on the older people and revealed that LRE-BFR has similar effects to CRE in terms of increased muscle strength and muscle mass [24]. Increased muscle mass has positive implications for obese individuals, such as body composition improvement and insulin sensitivity [25]. In an 18-week study, LRE-BFR was able to reduce the PBF, fasting plasma glucose (FPG) and fasting insulin (FINS) but increased ADP [25]. Research on the application of LRE-BFR in SO management is lacking, but LRE-BFR is beneficial to the musculoskeletal system and body composition of individuals with sarcopenia and those suffering from obesity. Thus, we speculate that LRE-BFR may be a potential exercise mode for older people with SO. However, little is known about the effects of LRE-BFR on the cardiovascular system, which suggests that physiological changes such as blood pressure and heart rate should be closely monitored when implementing interventions for older people with SO, who are at risk of CVD.

Therefore, this study has the following objectives: (1) to comprehensively evaluate the efficacy of a 12-week CRE and LRE-BFR intervention in improving the body composition, muscle strength, physical performance, haematological parameters, CVD risk factors and quality of life of older people with SO and (2) to compare the efficacy of CRE and LRE-BFR and explore their potential mechanisms.

## Methods and analysis

### Study design

**Fig. 1:**
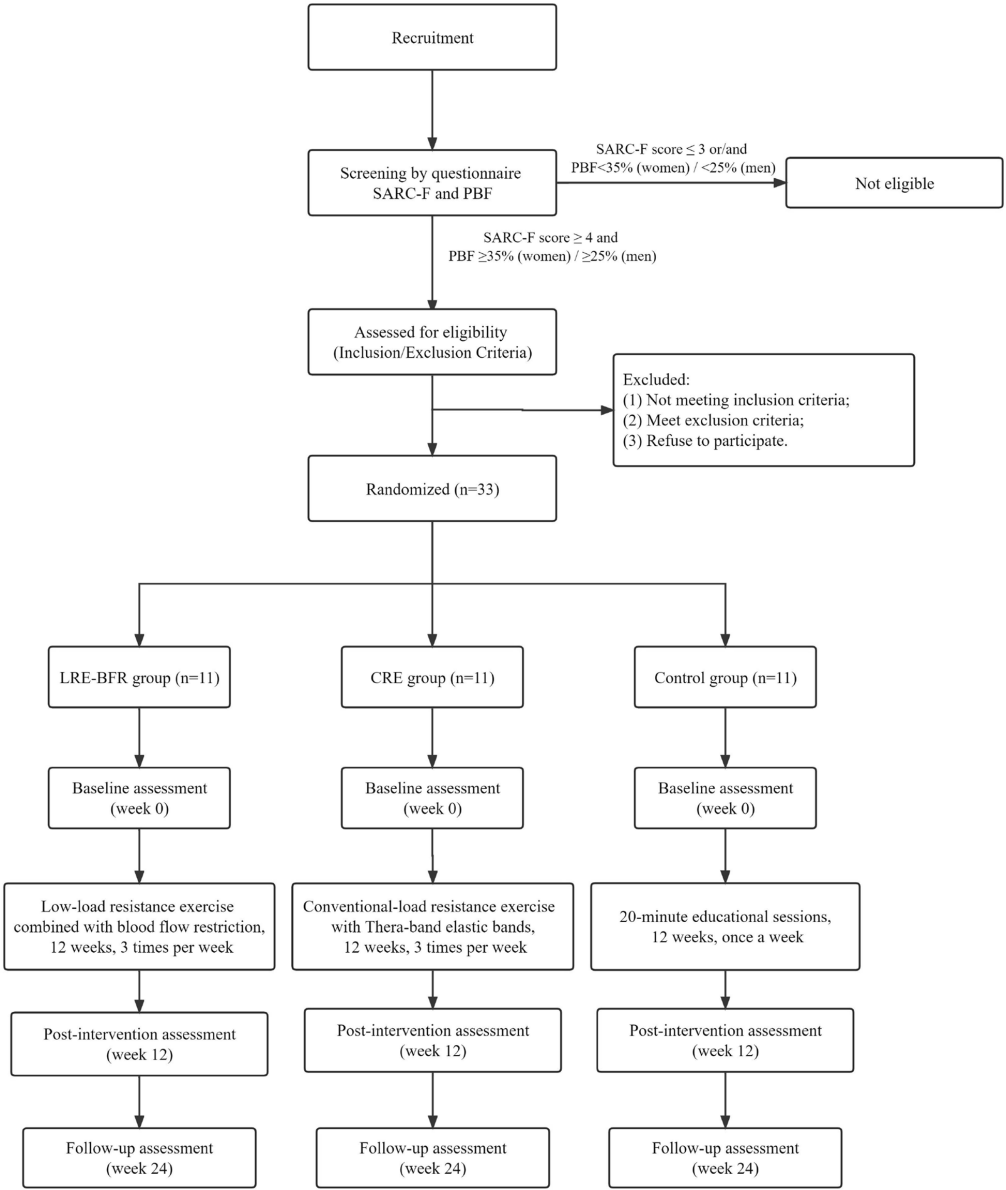
Flow chart showing the participant recruitment, intervention and assessment process. PBF: percentage of body fat; LRE-BFR: low-load resistance exercise with BFR; CRE: conventional-load resistance exercise

This work is a 12-week assessor-blinded randomised clinical trial (RCT) that will be conducted thrice a week, with two intervention groups and one control group. This study was registered on the Chinese Clinical Trial Registry, with registration number ChiCTR2300067296. The experimental flow chart is shown in Fig. 1, and the intervention and assessment schedules are presented in Table 1. The 2013 Standard Protocol Items for Randomized Trials checklist [26] is available in Additional file 1.

**Table 1:** The timetable for participant recruitment, intervention, and assessment

Measurement	Before (week 0)	Baseline (week 0)	Intervention (week 1-week 12)	End of intervention (week 12)	Follow-up (week 24)
Enrollment					
Recruitment and screening	X				
Information consent	X				
Allocation		X			
LRE-BFR			X		
CRE			X		
CG			X		
Primary outcomes					
PBF		X		X	X
KES		X		X	X
Secondary outcomes					
Body composition		X		X	X
Anthropometric measurements		X		X	X
Muscle strength of upper limbs		X		X	X
Physical performance		X		X	X
Hematological parameters and CVD risk factors		X		X	X
Quality of life		X		X	X
Others					
Participants' compliance			X	X	X
Adverse events			X	X	X
Reasons for dropout			X	X	X

*LRE-BFR* Low-load resistance exercise with BFR, *CRE* Conventional-load resistance exercise, *CG* Control group, *PBF* Percentage of body fat, *KES* Knee extension strength, *CVD* Cardiovascular disease

### Study setting

Recruitment will be conducted in four communities in the Chongming District, Shanghai, China, from 1 October 2023 to 1 December 2023. According to the established inclusion and exclusion criteria, 33 participants will be screened for the group interventions. All the participants will be assessed at three time points: before the intervention (week 0), at the end of the intervention (week 12) and after the intervention (week 24).

### Inclusion criteria

- 1. Individuals aged ≥ 65 years.
- 2. Individuals who meet the SO diagnostic criteria.

### Sarcopenia

Appendicular skeletal muscle mass index (ASMI) measured via bioelectrical impedance analysis (BIA): < 7.0 kg/m^2^ in men and < 5.7 kg/m^2^ in women; and handgrip strength (HS): < 28 kg in men and < 18 kg in women; and/or 6-m gait speed (GS) < 1 m/s (according to the Asian Working Group for Sarcopenia [AWGS] 2019) [27]

### Obesity

PBF ≥ 35% for women and ≥ 25% for men (World Health Organization [WHO] criteria) [28]

- 3. Individuals with no regular exercise habit (do not engage in PA at least 3 days/week, 30 min/day, of moderate intensity, for at least 3 months) [29]
- 4. Voluntary participation and a signed informed consent form.

### Exclusion criteria

- 1. Individuals with absolute contraindications to exercise, such as deep vein thrombosis and/or coagulation disorders
- 2. Individuals with a cognitive impairment, hearing impairment or visual impairment that will prevent normal communication with the researchers; cognitive impairment will be assessed using the Mini-Mental State Examination [30], with the score interpreted according to the participant’s literacy level; a score of ≤ 17 for illiterates, ≤ 20 for elementary school graduates and ≤ 24 for middle school graduates indicates cognitive dysfunction.
- 3. Individuals that will experience difficulty completing the assessment and/or exercise interventions, such as those with a musculoskeletal disorder (e.g. fracture and/or dislocation), CVD (uncontrolled hypertension with systolic blood pressure [SBP] > 200 mmHg or diastolic blood pressure [DBP] > 110 mmHg, uncontrolled dyslipidaemia with total cholesterol [TC] > 220 mg/dl, history of stroke, acute myocardial infarction, angina pectoris and uncontrolled ventricular tachycardia) or disability (e.g. loss of hands or feet).
- 4. Individuals with an infectious skin disease and/or unhealed and/or festering wounds
- 5. Individuals who have been taking the following medications regularly for the past 3 months: antiplatelet medications (e.g. aspirin, cilostazol, Aggrenox, cilostazol, ticlopidine, eptifibatide and tirofiban), analgesic medications and anticoagulant medications.

### Termination criteria

- 1. Individuals who will withdraw from the study at any time.
- 2. Individuals who will experience serious adverse effects during or after the exercises and will be unable to continue their participation.
- 3. Individuals who will lose contact during the exercise interventions and/or follow-up period.

### Recruitment

The participants will be recruited through the following four ways:

- 1. During annual physical examinations in four communities in the Chongming District, Shanghai, China.
- 2. During educational lectures on SO that will be conducted in four communities in the Chongming District to attract potential participants.
- 3. During home visits to older people who do not participate regularly in their annual physical examination, in cooperation with community workers.
- 4. The research proposal posted on the community bulletin board.

### Screening

The participants will be determined through two steps.

Step 1: All the recruited older people will be screened using the SARC-F questionnaire and PBF. Only those who obtain a SARC-F questionnaire score of ≥ 4 and with a PBF of ≥ 35% (women) or ≥ 25% (men) will proceed to the second step.

The SARC-F questionnaire includes five components: strength, assisted walking, rising from a chair, stair climbing and falling [31]. The questionnaire has a total score ranging from 0 (best) to 10 (worst), and a total score of ≥ 4 is considered to be at risk of sarcopenia [27].

Step 2: The final participants of the study will be assessed further using the inclusion and exclusion criteria.

### Randomisation and blinding

The final participants will be randomly assigned to an LRE-BFR, CRE or control group, with a 1:1:1 ratio. To ensure the allocation concealment, the randomisation sequence will be generated after the baseline assessment by a statistician who is not involved in the study, using SPSS version 26 (SPSS, Inc., USA), and stratified by gender and age to achieve a balanced distribution, with as many males and females in each group as possible. An electronic file of the random numbers and grouping will be encrypted by the statistician and sent to another external researcher to store the information. The information will be given to the therapist in a sealed envelope. All the outcome assessors, statisticians and data managers involved in this study will be blinded to the group assignment prior to the completion of the statistical analysis. Given the nature of this study, the participants and therapist cannot be blinded to the group assignment.

### Interventions

#### Group protocols

Before the formal interventions, the participants in the LRE-BFR and CRE groups will undergo 1RM testing and be taught how to use the Borg rating of perceived exertion (RPE) scale ranging from 6 to 20 to determine the target resistance load, and the RPE scores during the intervention will be recorded. The exercise intensity will be set based on the TheraBand force–elongation table to select the appropriate elastic band, which is reflected in the elastic band colour, from yellow to red to green. Limb occlusion pressure (LOP) testing will be performed in the LRE-BFR group. The participants’ attendance and occurrence of any adverse effects during the exercise interventions and follow-up period will be recorded by a dedicated investigator. If a participant misses a planned exercise intervention, they will make up the session at another time during the same week. All the participants will have three preintervention sessions to familiarise themselves with the exercise flow. The intervention protocols for the three groups are presented in Table 2.

**Table 2:** Details of the three groups of protocol

Weeks	Group	Specific intervention content	Sets	Repetitions	Interval rest time between sets (s)	Intensity	Frequency (times/week)
1–4	LRE-BFR	Biceps bend, seated row, seated knee extension	4	30,15,15,15	30	20% 1RM	3
	CRE					60% 1RM	
	CG	Education session (no intervention)	/	/	/	/	1
5–8	LRE-BFR	Biceps bend, seated row, seated knee extension	4	30,15,15,15	45	25% 1RM	3
	CRE					65% 1RM	
	CG	Education session (no intervention)	/	/	/	/	1
8–12	LRE-BFR	Biceps bend, seated row, seated knee extension	4	30,15,15,15	60	30% 1RM	3
	CRE					70% 1RM	
	CG	Education session (no intervention)	/	/	/	/	1

*LRE-BFR* Low-load resistance exercise with BFR, *CRE* Conventional-load resistance exercise, *CG* Control group, *1RM* One-repetition maximum

#### LRE-BFR

The participants will undergo a 30-min LRE-BFR (with TheraBand elastic bands) intervention three times a week for 12 weeks. The 30-min intervention will consist of a 5-min warm up, 20-min LRE-BFR and 5-min relaxation period. The exercise load will be set to 20%–30% of 1RM, based on Cook et al. [32] and the ACSM [33]. Each session will be composed of four sets of 30–15-15–15 min, with a rest of 30–60 s between sets. The three resistance movements will be bicep bends, seated rows and seated knee extensions, and each movement must be held for 3 s until the end.

Nylon cuffs inflated with an inflatable pump (TD312 Hokanson™, Bellevue, USA) will be tied to the proximal end of the two lower limbs of a participant at 50% LOP [34]. The individualised LOP will be determined before the start of the intervention. A vascular doppler probe (DV-600, Marted, São Paulo, Brazil) will be used to detect the participants’ lower limb tibial artery whilst resting in a horizontal position. The pressure at which the nylon cuff will be inflated until the arterial auscultation disappears will be the LOP [35]. The nylon cuff should be continuously inflated and not cause discomfort throughout the intervention.

#### CRE

The participants will undergo a 30-min CRE (with TheraBand elastic bands) intervention three times a week for 12 weeks. The 30-min intervention will consist of a 5-min warm up, 20-min conventional-load elastic band intervention (60%–70% of 1RM) and 5-min relaxation period. The number of repetitions, rest duration and movements will be the same as those in the LRE-BFR group.

#### Control group

The participants in the control group will not be given any exercise intervention but will participate in a weekly 20-min health education session on the meaning of SO, its risk factors and its adverse health outcomes. The International Physical Activity Scale Questionnaire-Short Form will be collected weekly to ensure that the participants are maintaining their daily PA level.

#### Nutritional intake

During the 12-week intervention and 12-week follow-up period, the researchers will require all the participants to maintain their daily dietary habits. The participants will be interviewed by professional researchers and community workers over the telephone regarding their diet (e.g. protein and fat intake at three meals a day), which will be recorded in a specific notebook. The frequency of the telephone interviews and monitoring will be 3 times a week (twice on weekdays and once on weekends), and the conversations will be recorded.

#### Outcome measurements

Measurements will be performed at the baseline (week 0), end of the intervention (week 12) and follow up (week 24).

### Primary outcomes

#### PBF

The PBF will be measured with a BIA instrument (Inbody S10, Korea), which is one of the assessment tools recommended by the AWGS 2019 owing to its noninvasive, inexpensive and easy-to-use nature and portability [27]. The participants must stand for more than 10 min before the measurement to redistribute the water inside their body, with their arms about 15º away from their trunk and feet, not barefoot, shoulder-width apart. The researcher will place eight contact electrodes on specific parts of their body (i.e. thumbs, index finger of both hands and inner and outer ankle of both lower limbs), and the participants must avoid touching any metal object other than the BIA instrument during the measurement. The BIA instrument will automatically measure the participants’ PBF.

#### Muscle strength of lower limbs

Lower limb muscle strength is reflected by the KES, which is assessed by the estimated 1RM. The standard 1RM test has exercise injury risks for older people [36]; therefore, the estimated 1RM test will be used to measure the exercise load. The exercise load will be measured in kilograms, with the participant sitting in an extensor chair equipped with a weight plate with an initial load of 45% of the body weight for women and 64% of the body weight for men [37, 38]. If the participant can perform knee extensions > 10 times with the initial weight, then the plate weight should be increased until the participant can repeat the exercise 10 times or less, at which the submaximal weight will be set as the weight of the plate. The following calculation formula will be used: estimated 1RM (kg) = submaximal weight (kg)/(1.0278—maximal repetitions*0.0278) [39].

### Secondary outcomes

#### Body composition

Body composition includes muscle mass (appendicular skeletal muscle [ASM] and ASMI) and obesity-related indicators (total fat mass [FM]). The ASM and FM will be measured directly through BIA, and the ASMI will be calculated with the following formula: ASMI (kg/m^2^) = ASM (kg)/height^2^ (m) [27].

#### Anthropometric measurements

The morphometric measurements will include the waist circumference (WC), hip circumference (HC) and waist-to-hip ratio (WHR). The researcher will measure the participant’s WC (circumference of the midpoint between the lowest rib point and upper edge of the iliac crest on a horizontal plane) and HC (the most prominent circle of the hip) whilst in a standing position, using a soft dermatome, then divide the WC by the HC to get the WHR.

#### Muscle strength of upper limbs

Changes in the upper limb muscle strength will be reflected in the HS. The measurement procedure is as follows: (1) the participant will hold a Jamar® Plus handheld grip dynamometer (Jamar Plus + Digital Hand Dynamometer; IL, USA) at a 90º elbow flexion whilst in a seated position, then (2) perform the grip on each hand three times at maximum strength. The maximum value will be recorded as the HS [27].

#### Physical performance

Physical performance will be assessed with the 6-m walk test and short physical performance battery (SPPB) test. We chose the SPPB and 6-m walk tests, because they are recommended by the AWGS 2019 [27]. In addition, a systematic review of 12 functional assessment tools for older people pointed out that the SPPB test has the highest validity, reliability and responsiveness scores [40]. For the 6-m walk test, the participants will be asked to walk 6 m at their daily GS, which will be timed by a researcher using a manual stopwatch, and the average result of at least two tests will be recorded as the final GS [27].

The SPPB test consists of three elements: the standing balance test, 4-m walk test and chair sit-to-stand test [41]. For the standing balance test, the participants will be asked to stand side by side, stand in semitandem and stand in tandem, following the researcher’s command, and the researcher will score the participants based on the duration. For the 4-m walk test, the participants will be instructed to walk 4 m at their regular walking speed. The test must be repeated and measured 2 times, and the shortest time will be recorded. For the chair sit-to-stand test, the participants must cross their arms in front of their chest whilst in a sitting position and repeat standing up and sitting down 5 times at their fastest speed. The exercise will be timed from the beginning of the sitting position to the standing position.

#### Haematological parameters and CVD risk factors

A professional nurse will collect each participant’s upper arm venous blood before the baseline (week 0), after the end of the intervention (week 12) and at the follow up (week 24). The participants will be asked to fast for 12 h before the blood collection. The blood samples will be sent to a laboratory for testing.

The following indicators will be determined via automated enzyme-linked immunosorbent assay: inflammatory factors (IL-6, CRP and TNF-α), hormones (IGF-1), growth factors (MSTN), adipose factors (LEP and ADP) and CVD risk factors (FPG, triglyceride [TG], TC, high-density lipoprotein cholesterol [HDL-C], low-density lipoprotein cholesterol [LDL-C] and FINS).

Insulin sensitivity will be expressed by the homeostasis model assessment of the IR (HOMA-IR), with a decrease in the HOMA-IR representing an increase in insulin sensitivity. The calculation formula is HOMA-IR = (FINS μU/mL*FPG mmol/L)/22.5 [42].

The other CVD risk factors (resting heart rate [HRrest], SBP and DBP) will be measured using an electronic blood pressure monitor (OMRON, U30, China).

#### Quality of life

The participants’ quality of life will be assessed using the Chinese version of the 36-item Short Form Health Survey Scale (SF-36), which has been confirmed as reliable and valid, with an overall Cronbach’s α coefficient of 0.943 and each dimension having a Cronbach’s α coefficient of > 0.70 [43, 44]. The SF-36 consists of 36 items covering eight domains (i.e. physical function, physical role, physical pain, general health, vitality, social function, emotional role and psychological health) [45]. The total score will range from 0 to 100, and the higher the score, the higher the quality of life.

#### Sample size

The sample size will be calculated using G*Power version 3.1 [46]. Clarkson et al. [47] indicated that the outcome that is most relevant to the effect of BFR is the KES; therefore, the KES will be used as the primary outcome indicator to calculate the sample size. The effect size f of the study will be determined as 0.65 based on a previous study [24]. The relevant parameters will be set, as follows: an alpha of 0.05, a power of 0.8 and an effect size of 0.65, with a total sample size of 27 participants (9 participants per group). The total sample size of the study will be set to 33 participants (11 participants per group), considering a 20% dropout rate.

### Statistical analysis

All the outcome data assessed in the study will be entered into a database via SPSS Statistics version 26 (SPSS, Inc., USA) for the statistical analysis. All the data will be expressed as the mean ± standard deviation or median. The categorical variables will be tested using the chi-square test, and comparisons between the groups will be conducted using one-way ANOVA or the Kruskal–Wallis test. The results will be described as 95% confidence intervals and regarded as significant at *P* < 0.05.

Differences in the outcomes over time between the LRE-BFR, CRE and control groups will be analysed with linear mixed models following the intention-to-treat principle. Missing data will be designated as randomly missing, and the data will be processed using maximum likelihood and linear mixed models.

#### Safety monitoring

We are professionally trained to fully supervise and guide the participants during the exercise intervention to reduce their risk of injury. We are obligated to report the occurrence of any adverse effects during the intervention to the local ethics committee and work together to find the best solution. The participants have the right to terminate their participation in succeeding experimental interventions.

#### Data management and monitoring

All the relevant data and information for this study will be recorded and stored using Microsoft Excel 365, including the participants’ sociodemographic characteristics and pre- and postintervention assessment data and the occurrence of any adverse effects during the intervention. To ensure the research confidentiality, all the computers and databases will be encrypted, and the participants’ names will be replaced with numbers. The paper and electronic versions of the data will be destroyed after 5 years.

The Data Monitoring Committee, which is independent of competing interests and sponsors, is composed of clinical experts, researchers, trialists and statisticians.

#### Patient and public involvement

The first time the public will participate in the study will be during the recruitment. The recruitment will be conducted during the annual community physical examination, educational lectures and home visits and through bulletin board promotion. The study results will be reported individually at the end of the follow-up period. The participants/public will not be involved in the design of the study and will be informed of the time required for the study before the start of the formal intervention. Then, the participants will be required to sign an informed consent form if they are willing to participate in the study.

#### Ethics and dissemination

The Ethics Research Committee of Chongming Hospital Affiliated to Shanghai University of Medicine and Health Sciences approved the study (approval No. CMEC-2022-KT-51) in December 2022.

The data of the participants to be collected are as follows: demographic information (i.e. name, age, gender, occupation, marital status and residence status), anthropometric measurements, body composition, muscle strength, physical performance, haematological parameters, quality of life, PA level and CVD risk factors. The participants will be required to give their signed informed consent. All the paper data will be stored in the project office, and the electronic version of the data will be encrypted and stored in the computer of Chongming Hospital Affiliated to Shanghai University of Medicine and Health Sciences.

Changes or developments in the study will be reported at www.chictr.org.cn.

## Discussion

To the best of our knowledge, this RCT will be the first to comprehensively assess and compare LRE-BFR, CRE and control groups in terms of their body composition (PBF, ASM, ASMI and FM), anthropometric measurements (WC, HC and WHR), muscle strength (KES and HS), physical performance (GS and SPPB), haematological parameters (IL-6, CRP, TNF-α, IGF-1, MSTN, LEP and ADP), CVD risk factors (FPG, TG, TC, HDL-C, LDL-C, FINS, HOMA-IR, HRrest, SBP and DBP) and quality of life (SF-36). Specifically, this RCT will be the first to use LRE-BFR for older people with SO. SO may cause complications [9] and have an all-cause mortality rate of up to 24% [48]; thus, clinical staff and researchers must pay attention to SO and find effective exercise modes to manage the condition.

Centner et al. [49] reported that LRE-BFR is a recommended exercise mode for older people with CRE contraindications but require urgent intervention. Sarcopenia is characterised by type II muscle fibre atrophy and a reduction in satellite cells [50]. Inadequate distal muscle oxygenation will result from BFR on the proximal limb, which will promote the accelerated recruitment of type II fast muscle fibres, which are less dependent on aerobic metabolism [51]. The higher the muscle mass, the faster the metabolism, and the more the calories consumed, which can undoubtedly contribute to fat loss [52]. The improvement of muscle mass and strength from LRE-BFR was reported to correlate with the mammalian target of rapamycin [53], MSTN [54] and muscle protein synthesis [53]. However, the studies mentioned above involved healthy older people [53], and studies on SO involving older people with inclusion body myositis have yet to be conducted [54]. Therefore, conducting a study on LRE-BFR involving older people with SO is necessary to clarify its efficacy and safety. The development of SO is closely related to haematological parameters such as inflammatory indicators, hormones, growth factors and adipose factors; thus, the changes in such parameters before and after the intervention may reflect the physiological mechanism of LRE-BFR.

This study has several strengths. Firstly, elastic bands as a mode of resistance exercise are more portable, cheaper and safer than weight machines [55]. Most weight machines may be difficult to use for novice exercisers. For example, the load of some weight machines may increase to 5–10 lbs at a time, which may lead to sprains in older people owing to the excessive load range and their diminished muscle adaptation [56, 57]. In addition, older people may drop barbells and weightlifting machines, which may cause fractures or induce severe effects.

Secondly, previous studies on LRE-BFR focused mainly on changes in muscle mass or strength in the lower limbs [23, 58, 59]. SO is a geriatric syndrome that can induce changes in whole-body composition, including a decrease in muscle mass and strength and an increase in fat, which are closely related to haematological parameters, CVD risk factors and quality of life [10]; however, such changes generally go unnoticed. Therefore, our study is comprehensive, because it will measure pre- and post-LRE-BFR changes in body composition, haematological parameters, CVD risk factors and quality of life to explore the application of LRE-BFR in the physiological mechanism of older people with SO.

Thirdly, the PBF may be more reliable than the body mass index (BMI) to identify obesity. In the early development of SO, external factors such as low PA and high-fat or high-sugar diets may lead to fat deposition in the abdomen and infiltration into the muscle [60]. As inflammation and IR increase, adipose tissue will gradually spread from the local to the systemic level and thus worsen SO [60]. Owing to the reciprocal masking effect of muscle loss and fat gain in the early progression of SO, many older people with a normal BMI may unknowingly have SO. Thus, the PBF may better characterise the progression of SO than the BMI.

Lastly, this study will evaluate the efficacy of LRE-BFR in reducing CVD risk factors to explore its implications on the cardiovascular health of older people with SO. Although LRE-BFR is a novel intervention strategy for managing the physical health of older people [50], evidence on its cardiovascular safety is lacking, especially for older people with CVD risk factors. An 8-year cohort study on people aged ≥ 65 years found that older people with SO have a significantly increased risk of CVD by 23% compared with older people with no SO [9]. Exploring the potential impact of LRE-BFR on cardiovascular health is essential if LRE-BFR is to be widely promoted among older people with SO.

Although BFR can offer new ideas for exercise interventions, limitations remain in its use. Existing studies focused little on the safety of LRE-BFR, especially for frail older people. The exercise prescription design of this study is based on the BFR exercise guidelines [61] and ACSM resistance exercise recommendations for older people [33], which are generalisable. However, whether it is an effective exercise prescription for older people with SO remains unknown. Cook et al. [62] observed that muscle fatigue induced by LRE-BFR (20%–40% of 1RM) is similar to or higher than that induced by high-load (80% of 1RM) resistance exercise. Although fatigue may stimulate an increase in muscle mass or strength, it may decrease the compliance of older people with SO. Therefore, we will constantly monitor the participants’ fatigue in our study and conduct timely adjustments of the exercise prescriptions, when necessary, to decrease the injury risk of the exercise interventions.

For older people with SO, LRE-BFR is a novel exercise mode whose efficacy has yet to be investigated. By comparing the effects of LRE-BFR and CRE, our study will contribute an easy-to-perform and efficient mode of exercise that will enable clinical staff and researchers to effectively manage older people with SO.

### Supplementary Information

**Additional file 1. **SPIRIT_Fillable-checklist.

## Data Availability

The datasets generated during the present study will be available from the corresponding author on reasonable request.

## Abbreviations

SO: Sarcopenic obesity
CRE: Conventional-load resistance exercise
LRE-BFR: Low-load resistance exercise combined with blood flow restriction
CVD: Cardiovascular disease
RCT: Randomised clinical trial
AWGS: Asian Working Group for Sarcopenia
WHO: World Health Organization
PBF: Percentage of body fat
IR: Insulin resistance
IL-6: Interleukin 6
CRP: C-reactive protein
TNF-α: Tumour necrosis factor-α
IGF-1: Insulin-like growth factor 1
LEP: Leptin
ADP: Adiponectin
MSTN: Myostatin
PA: Physical activity
ACSM: American College of Sports Medicine
1RM: One-repetition maximum
BFR: Blood flow restriction
HS: Handgrip strength
KES: Knee extension strength
ASM: Appendicular skeletal muscle
FPG: Fasting plasma glucose
FINS: Fasting insulin
ASMI: Appendicular skeletal muscle mass index
BIA: Bioelectrical impedance analysis
GS: Gait speed
BMI: Body mass index
SBP: Systolic blood pressure
DBP: Diastolic blood pressure
TC: Total cholesterol
RPE: Rating of perceived exertion
LOP: Limb occlusion pressure
FM: Total fat mass
WC: Waist circumference
HC: Hip circumference
WHR: Waist-to-hip ratio
SPPB: Short physical performance battery
TG: Triglyceride
HDL-C: High-density lipoprotein cholesterol
LDL-C: Low-density lipoprotein cholesterol
HOMA-IR: Homeostasis model assessment of insulin resistance
HRrest: Resting heart rate
SF-36: 36-Item Short Form Health Survey Scale
